# The FEDBD plasma’s quantitative investigation of skin parameters: Skin elasticity, thickness, density, tissue oxygenation, perfusion, and edema

**DOI:** 10.1016/j.heliyon.2023.e23386

**Published:** 2023-12-12

**Authors:** Parisa Charipoor, Mohammad Ali Nilforoushzadeh, Mohammadreza Khani, Maryam Nouri, Erfan Ghasemi, Mohammad Amir Amirkhani, Mohammad Eftekhari, Babak Shokri

**Affiliations:** a Laser-Plasma Research Institute, Shahid Beheshti University, G.C., P.O. Box, 19839-6941, Tehran, Iran; b Skin and Stem Cell Research Center, Tehran University of Medical Sciences, Tehran, Iran; c Skin Repair Research Center, Jordan Dermatology and Hair Transplantation Center, Tehran, Iran; d Physics Department of Shahid Beheshti University, G.C., P.O. Box, Tehran, 19839-6941, Iran

**Keywords:** Cold plasma, Floating electrode dielectric barrier discharge (FEDBD), Skin rejuvenation, Thickness, Density

## Abstract

This study used the FEDBD plasma device for skin rejuvenation in animal samples. There were two groups of six male Wistar rats. Before starting the treatment, immediately after the treatment, the fourth week, and the tenth week of follow-up, biometric tests were performed, including moisture level, evaporation from the skin surface, erythema and melanin, skin elasticity and firmness with an MPA9 device and cutometer. The thickness and density of the epidermis and dermis, an essential indicator in rejuvenation, were evaluated with a skin ultrasound device. Also, the level of oxygen, perfusion, and interstitial water (edema) was checked using a Tivita tissue hyperspectral camera at a depth of 6 mm of the skin.

## Graphical abstract

**Figure:**
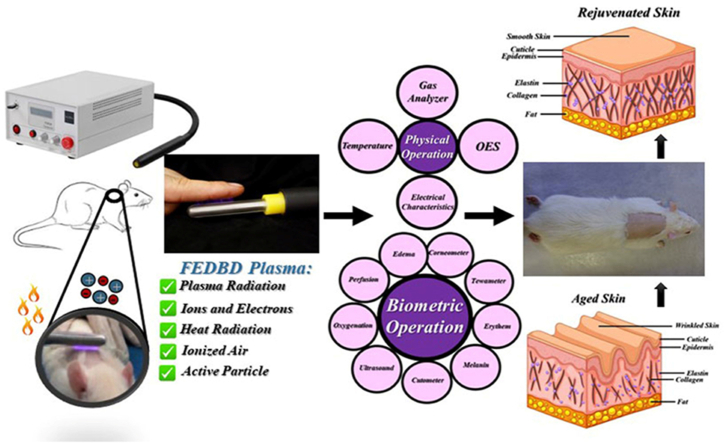

## 1 Introduction

The skin is the body's biggest organ. It governs various specialized functions due to its position in the interaction between the interior and exterior components of the body. Achieving biological and cosmetic objectives as well as preserving skin integrity against external substances like microbes and dehydration are crucial [1]. An age-related loss in performance during physiological and health-related processes is known as aging. Age and heredity are two external and internal variables that contribute to the multifaceted process of skin aging. Clinical signs of intrinsic aging include fine wrinkles, dry skin, color changes, and loss of suppleness. It is more noticeable in skin that has been shielded from the sun. From a biological perspective, greater skin thinning develops over time as a result of the delayed regeneration of the epidermis brought on by a decline in the ability of keratinocyte stem cells to proliferate, a decrease in the synthesis of collagen, and the depletion of epidermal stem cell reservoirs. Women's skin ages more quickly after menopause as a result of lower estrogen levels, so this has significant effects on how they look [2].

Signaling between skin fibroblasts and keratinocytes is required for tissue homeostasis in both normal and pathological circumstances [3]. The interaction between cell groups decreases with age, with keratinocytes and fibroblasts interacting much less. As a result, it affects fundamental stem cell growth and makes aged skin more brittle. Langerhans cells also diminish, which disrupts the skin's immunological system [4].

Chemical and physical procedures have been used to treat skin in recent years in an effort to renew it, remove old cells from it, or neutralize its effects and avoid negative repercussions, particularly with regard to the look of the skin. Rejuvenating procedures include the use of neuromodulators, filler injections, lasers, chemical peels, microdermabrasion, LED lights, and lasers. By physically eliminating the skin's outer layers, these light sources rejuvenate and invigorate the skin by accelerating the skin cells' metabolism [5,6].

Non-surgical energy-based treatments have grown in popularity recently. Plasma medicine is a new and creative area that blends plasma physics with clinical medicine. The interaction of particular plasma components with structural features and the function of live tissue cells is the focus of contemporary plasma medicine research. Plasma skin rejuvenation is a relatively recent technique that has been offered as an energy-based skin rejuvenation treatment that operates on many skin levels. It makes the skin acidic, moisturizes the skin's outermost layer, and clears the stratum corneum of pollutants. By weakening or rupturing the skin barrier, reactive oxygen and nitrogen species (RONS) generated by plasma can also permeate the skin and improve the absorption of other molecules, including medications and lotions. After reaching the skin, RONS can directly impact skin oxidation at the molecular level and initiate cell metabolism and communication. Increased skin oxygenation, vascular stimulation, extracellular matrix regeneration, fibroblast stimulation, and collagen production are all brought on by cold atmospheric plasma therapy and are crucial for skin rejuvenation.

Owing to a study, the discharge plasma of the floating electrode's dielectric barrier successfully kills melanoma skin cancer cells. With a high therapeutic dosage (15 s or more at 1.4 W/cm2 plasma therapy), floatated electrode dielectric barrier discharge (FE-DBD) plasma kills melanoma skin cancer cells by necrosis. Cell necrosis was not found at low doses (5 s at 0.8 W/cm2 plasma treatment), however melanoma cancer cells exhibited apoptotic activity. First, the treated cells stopped growing and perished in large numbers 12–24 h later, but the untreated cells continued to develop and multiply [7]. Similar to a different study, FE-DBD plasma may be used directly to coagulate blood and sterilize live tissue without causing damage or pain [8]. Acne is a form of skin illness that is an inflammatory disease of fat follicles that is caused by a variety of reasons. Vasin et al. established the efficiency of the DBD plasma device in treating acne vulgaris. In addition, 31 acne patients were treated with plasma once a week for six weeks, and 75 % of the lesions improved. There were also just a few minor negative effects, such as dry skin [9]. Five patients with 17 lesions each had actinic keratosis treated with DBD plasma by Friedman et al. After one month of therapy, nine lesions were eliminated and three greatly improved, resulting in a 70 % improvement in the complication and 53 % clearing of the skin. There were no reports of blisters or post-treatment irritation, and the procedure was entirely painless [10]. A comparison of the plasma skin regeneration system (PSR) and carbon dioxide laser on animal samples revealed that new collagen produced at the dermis and epidermis junction. The plasma approach revealed no signs of a wound, and the skin irritation subsided after six days. According to the findings of this study, there was a considerable reduction in wrinkles and hyperpigmentation [11]. A group studied the effect of FE-DBD cold plasma and vitamin C lotion on skin regeneration in 2021. For six weeks, Wistar rats were treated three times each week. The findings of this study reveal a rise in collagen levels after drinking plasma alone, as well as an accelerated skin response after consuming vitamin C cream in conjunction with plasma therapy. The thickness of the epidermal layer rose after utilizing high-voltage plasma, showing the skin's suppleness. This study demonstrates that plasma technology and vitamin C ointment have a good influence on skin renewal measures [12].

The current study looked at the efficacy of a floating electrode dielectric barrier discharge device on skin rejuvenation in animal samples. So yet, few investigations in this sector have been completed, and thorough quantitative measurements have not been performed. The depth of penetration and its effect on the underlying tissues were investigated. Optical spectroscopy was used to detect the existence of active species. Skin temperature was assessed to determine the thermal impact of plasma. In addition, the potential negative effects of FE-DBD plasma therapy were assessed using various analyses at various time intervals. The MPA9 gadget monitored many skin rejuvenation parameters, including skin moisture level, evaporation from the skin surface, melanin level, and erythema. The CUTOMETER instrument was also used to assess skin elasticity and firmness. Finally, the thickness of the epidermis and dermis, as well as the density of the epidermis and dermis, were evaluated using a skin ultrasound machine.

## 2 Materials and methods

### 2.1 Animals

The effectiveness of the FE-DBD plasma device was investigated on 12 three-month-old male Wistar rats (∼200 g) purchased from Pastor Laboratories (Tehran, Iran). All rats were kept in separate cages under standard conditions (12 h of darkness and 12 h of light, easy access to water and food, proper temperature, and ventilation). The desired number of mice was determined based on statistical consultation. The number of samples was 12 Wistar rats in two groups of six.

- The first group was subjected to plasma processing with a power of 3.3 W for 6 min.
- In the second group, the control group, no intervention was performed on rats.

The National Institute approved all animal procedures for Medical Research Development (NIMAD) of the Tehran University of Medical Sciences (protocol number IR. TUMS.AEC.1400.050).

The in-vivo study was reported in keeping with the standards for reporting experiments published in ARRIVE Animal Research: Reporting of In-vivo Animal Experiments [13]. After the experiments were done, all of the mice were sent to the Shahid Beheshti University Faculty of Biology to live.

### 2.2 Sample preparation and plasma processing steps

For plasma processing, rats must be anesthetized with 10 % ketamine and 20 mg/ml xylazine solution (Bremer pharma GMBH Warburg, Germany). The anesthetic compound must be injected intraperitoneally. After anesthetizing, the hair on their backs near the neck was removed. Then, using the probe of the plasma device FE-DBD of the specified area was swept, and the whole desired area was processed by moving the probe back and forth. Also, the time to do this work was 6 min, and processing times were considered for two sessions per week for one month. For applied plasma in an optimal state, the frequency of 21 KHz and the power of 3.3 W were considered, and the probe of the device was placed at a distance of 2–3 mm from the target surface, and all the tests were performed under these conditions.

**Fig. 1:**
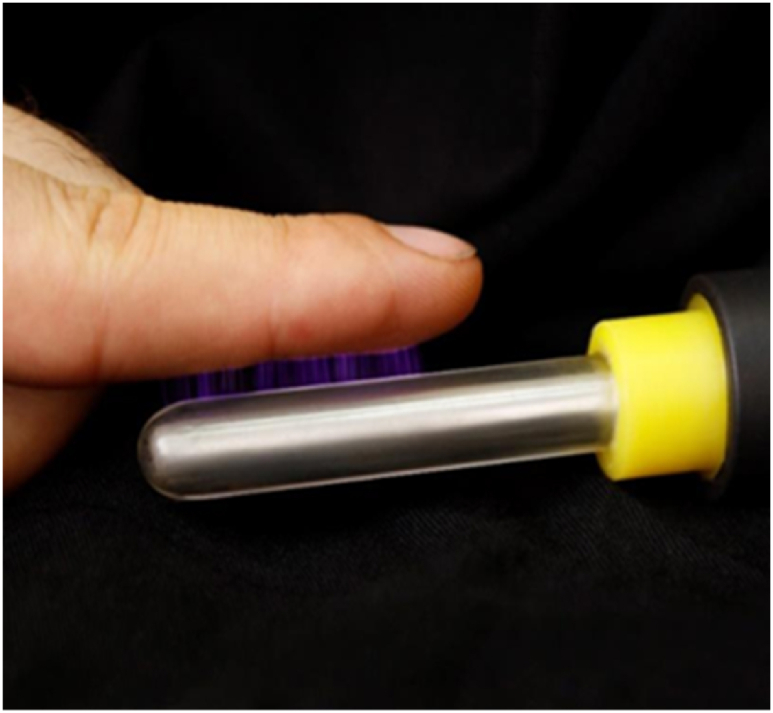
FE-DBDS surface treatment by FE-DBD device.

A uniform plasma by its probe (Fig. 1) is created to process the surface, and the skin of the animal (or human) body acts as the second electrode, and the plasma is applied directly to the surface.

### 2.3 Temperature evaluation

To determine the skin temperature changes, the temperature of the place under plasma treatment was recorded before the start of the treatment and at the second, fourth, and sixth minutes using a FLIR-E4 (Uncooled microbolometer) digital thermal camera with an image quality of 80*60 pixels and a thermal sensitivity of 0.15° Celsius.

### 2.4 Measuring and recording voltage

The electrical behavior of plasma and voltage changes in different intensities were evaluated using a high voltage probe (P6015A, 1:1000) and oscilloscope device DPO3012 (Tektronix, United States) by specifications including 100 MHz and 2.5 GS/S. This probe measures DC voltage up to 20 KV and AC voltage up to 40 KV.

### 2.5 Skin biometric

To investigate the possible side effects of plasma treatment, the samples were subjected to skin analysis before processing and immediately after processing, the fourth and tenth weeks. The Multi Probe Adapter (MPA-9) Courage + Khazaka electronic GmbH (Köln, Germany) device measured some skin characteristics. This device includes various probes to check skin characteristics, including moisture level, skin surface evaporation rate, brightness, erythema, and melanin, which were tested on several samples. A Corneometer CM825 probe (Courage + Khazaka electronic GmbH Germany) was used to measure skin hydration. In this method, the probe head was placed vertically on the desired skin surface, and the measurement was made according to the pressure of the spring inside the probe. The measurement starts while the probe is in contact with the skin, and its amount is shown on the screen. A transepidermal water loss (TEWL) TM300 probe was used to determine the amount of skin water leaving or evaporating from its surface. The tewameter probe was placed vertically on the desired skin. The measurement started by pressing the built-in button, and the minimum deviation was shown on the screen. A Mexameter MX18 probe was used to measure melanin and skin erythema. There are two sensors in the mexameter, one of which has three lights with a wavelength of green 568 nm, red 660 nm, and infrared 780 nm, which sends them to the skin.

To measure, the probe was placed vertically on the skin's surface by the operator with gentle pressure, and the value of melanin and erythema, redness caused by irritation, was shown on the screen. The skin's degree of stiffness and elasticity was measured using Cutometer Dual MPA 580 by suctioning the skin into the probe. This instrument assesses the skin's resistance to negative pressure (stiffness) as well as its capacity to recover to its original position (elasticity). It contains three indices: R2, R5, and R7, where R2 represents mechanical force resistance vs capacity to recover. R5 represents the elastic component of the suction phase vs the instant recovery in the relaxation phase, whereas R7 represents the immediate recovery following suction. When the cutometer probe is placed vertically on the target skin, it pulls in and releases it, and the degree of skin tightness is shown on the screen.

### 2.6 Skin scanning

DUB-Skin Scanner was used to measure skin, epidermis, dermis thickness, and skin density.

### 2.7 Evaluation of skin texture

TIVITA Tissue (Diaspective Vision, Pepelow, Germany) was utilized to examine skin tissue features over a wide range of wavelengths (500–1000 nm). Tissue oxygenation in surface layers (StO2), perfusion in deeper skin portions 4–6 mm, tissue hemoglobin index (THI), surface perfusion hemoglobin, and tissue water index (TWI) were among the metrics collected by this device using software algorithms.

### 2.8 Optical radiation spectroscopy

In this study, to determine the type and intensity of active species in plasma, the AVANTES spectrometer model AvaSpec-ULS 3648-USB2 with a resolution of 0.6–0.7 nm was used to determine the electron density of the plasma radiation spectrum. This spectrometer can detect wavelengths of 200–11000 nm.

### 2.9 Ozone detector

The ozone detector model SKY2000–O3 was used in the measurement range of 0.1–1000 ppm to detect the concentration of ozone gas. The gas concentration unit is ppm, and its displacement rate is mg/m^3^.

### 2.10 Statistical analysis methods

The collected data for different stages of the research were analyzed with the help of SPSS version 26 software. In all cases, the significance level of the tests was considered to be 0.05, and the error up to 0.05 was acceptable and negligible. The data was measured in two control groups and 3.3 W. Four times, including before processing, immediately after processing, the fourth week, and the tenth week after the experiment, from the analysis of repeated measurements after checking the presuppositions of the test were used. Then, in the significant tests, the LSD post hoc test was used to compare two by two and find meaningful comparisons. Also, using the Kolmogorov-Smirnov test, the normality of the residuals of the mentioned statistical model was investigated. Finally, using bar graphs, individual groups were compared, and then groups were compared in pairs to illustrate the results and conclusions as much as possible. For more accessible conclusions, significant points were marked with *** on the graphs. Also, to determine the significance, the time-dependent test was used in the single-group graphs, and the time-independent test was used in the two-group graphs.

## 3 Results

### 3.1 Physical analysis of FEDBD plasma

**Fig. 2:**
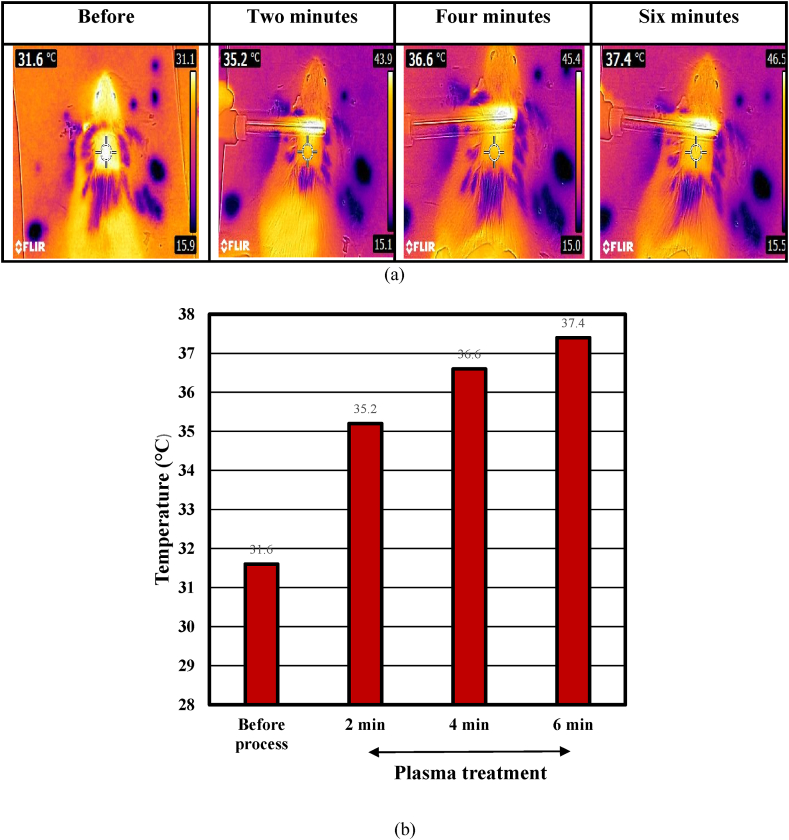
The image belongs to a) the temperature range of the skin surface and b) the graph of temperature change.

Temperature. To check the temperature of the plasma on the mouse skin over time, its temperature was measured and recorded before processing at 2, 4, and 6 min. The camera was placed perpendicular to the surface of the mouse skin at a distance of 30 cm, and the temperature generated by the plasma was recorded. The temperature range of the skin surface at the power of 3.3 W is shown in Figure (2 a), and the graph of temperature changes in 6 min is shown in Figure (2 b).

Due to the heat transmission of the gas to the skin, plasma quickly warms the skin after departing and transferring it to the treatment location. Heat shock is caused by the heat action of plasma on the skin. The dermal fibroblasts are stimulated by the temperature shock, which leads to protein production and collagen synthesis [14]. Increasing the skin temperature to 37–38.5° Celsius stimulates keratinocyte growth and aids in tissue repair. In general, tissue temperature should not be higher than 40° Celsius. Temperatures between 40 and 50° Celsius cause local hyperthermia of the cell membrane and molecular structural breakdown. Cell necrosis can also result [15].

Exposing the skin to a temperature of 45° Celsius leads to denaturation, loss of function, and structural changes of proteins and causes skin burns. The plasma temperature on the skin in 6 min with a power of 3.3 W is below 40° Celsius, which can be a suitable thermal shock for the stimulation and proliferation of fibroblast cells. Also, using a high spectral camera showed that this temperature increases blood supply and oxygenation in the skin and does not harm the skin.

**Fig. 3:**
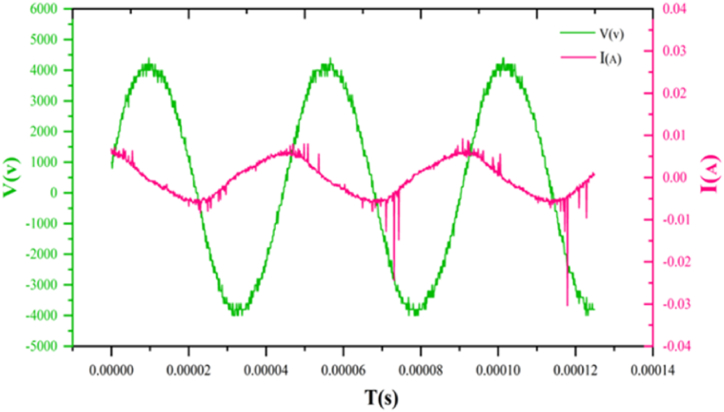
Voltage and current waveforms applied by the power supply.

Voltage. The curve of voltage and current changes over time determines the electrical behavior of plasma. The FE-DBD plasma device is a type of radiation discharge that, when the plasma is turned on at high peaks, has current peaks that do not change much but change over time due to voltage changes. The electrical characteristics of the FE-DBD plasma device were investigated and measured by a high-voltage probe, a current probe, and an oscilloscope. The peak-to-peak voltage for 3.3 W power was 4 kV for low and 4.5 kV for high voltages. Also, the frequency of this device was 21 kHz (Fig. 3). Calculating the device's power through the voltage and current diagram or the Lissajous curve has no significant difference, and the error percentage is low [16].

**Fig. 4:**
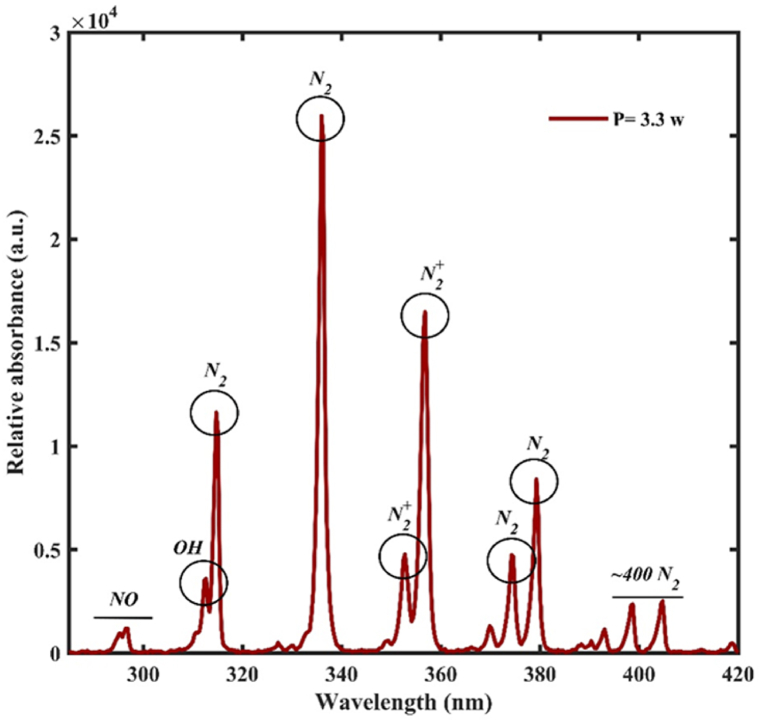
FE-DBD plasma emission spectrum.

Spectroscopy of optical emission. The intensity of light emitted by the plasma is measured in plasma spectroscopy. At 3.3 W of power, the kind and intensity of the active species created in the plasma were determined in Fig. 4. Because of the quantity of these species in the air (78 % nitrogen), the majority of the species created in this plasma are active nitrogen species. The wavelength range of the OES spectrum is 280–420 nm. The FE-DBD plasma device's optical emission spectroscopy revealed the emission of NO species at 297 nm, OH at 309, N2, and N2+ at wavelengths (315, 337, 354.7, 358.08, 375.4, 380, 406) [12,17,18].

**Ozon detector.** Ozone is an unstable molecule and a strong antioxidant that destroys microorganisms without any resistance and is produced naturally by neutrophils that work in the body's defense mode. Ozone gas has properties such as increasing metabolism, accelerating the healing process, reducing the speed of aging, improving blood circulation, and increasing physical immunity. It also has antioxidant properties, reducing oxidative stress due to reducing flare-ups and stimulating microcirculation, which increases tissue oxygenation capacity and has the power to regenerate tissue due to its immunological function. Therefore, it helps stimulate and multiply fibroblasts, cells responsible for producing collagen, glycosaminoglycans, and proteoglycans. These are the cell matrix's main components and are responsible for strengthening the skin [19]. The concentration of ozone gas produced by a plasma device with a power of 3.3 W was measured and recorded. The tube connected to the sensor was placed on the surface of the mouse's skin, and then, by moving the probe of the plasma device, the concentration of this gas was recorded in 6 min. The ozone concentration during processing was 27 ppm and zeroed 3 cm from the target surface. Also, the amount of ozone on the processed surface was zero after processing. To further ensure the safety of the patient's and the operator's respiratory system when working with the device, it is better to turn on the ventilation.

**Carbon monoxide.** Carbon monoxide gas is dangerous, and breathing large amounts of it can be fatal. CO interacts to hemoglobin in the lungs to generate carboxyhemoglobin (COHb). When the concentration of CO in the blood is high, the oxygen-carrying capacity of the blood declines, resulting in tissue hypoxia and, in some cases, permanent damage. The proportion of carboxyhemoglobin in the blood should be less than 10 %; up to 1000 ppm of generated gas is safe for this purpose [20].

The amount of CO produced in FE-DBD plasma processing was measured within 6 min using a PTM600 series portable gas analyzer. At a power of 3.3 W, CO produced was 2.3 ppm, which is entirely safe. A low concentration of CO can have beneficial effects on the health of the body. This molecule is stable and does not produce toxic side products. It is a natural signaler that can play an important regulatory role as a messenger in biological systems. It has anti-inflammatory, vasodilating and anti-proliferative properties [21]. Co-produced by plasma, it reacts with particles on the skin and cleans it of fats and impurities. In addition, it causes more blood supply and oxygenation in the skin, and then the proliferation of fibroblasts in the skin is stimulated, and the synthesis of collagen increases.

**Nitrogen monoxide:** Each plasma parameter that interacts with skin tissue has a unique set of biochemical, biophysical, and biological impacts. Several types of cells, including skin cells, create NO in the body. The active form of NO, as well as thermal shock, have a larger role in skin regeneration [22,23]. This molecule functions as a messenger in cell communication and is crucial in the neurological, circulatory, and immunological systems of the body [24].

NO, which is formed by the interaction of plasma with air, has been shown to speed up the healing of diabetic wounds [25]. According to additional research, NO normalizes blood circulation, which decreases inflammation while increasing bacterial phagocytosis and fibroblast growth. As a result of the increased fibroblast cell count, collagen protein synthesis rises, resulting in repair and, hence, rejuvenation.

Suschek et al. demonstrated that the vasodilator action of NO in plasma can improve skin microcirculation without creating negative effects. Inflammatory cells enter the skin, produce growth factors and different cytokines, and drive cell proliferation, including fibroblasts, which eventually aids skin renewal by multiplying collagen formation [24].

**Safety.** Ozone is the most critical air pollutant with harmful effects on the respiratory system of humans and animals. Due to its antibacterial effect, it is used to disinfect air and water. The odour threshold for ozone is 0.02 ppm or 0.04 mg/m3. In this study, mouse skin was used as a simulation for human skin and treated with FEDBD plasma. The average ozone concentration produced was 27 ppm. After 120 s, the concentration reached 20 ppm, which may be due to the higher temperature of the plasma, which does not allow the initiation of chemical reactions that lead to the formation of ozone. The half-life of ozone decreases rapidly with increasing temperature [26]. No ozone content in ppm was recorded at a distance of 3 cm from the target surface, but for more certainty, it is better to do this under the hood.

CO interacts with blood hemoglobin ten times more than oxygen, reducing the oxygen-carrying capacity of the blood and disrupting the oxygen delivery to the tissue. Morbidity and mortality are induced by hypoxic conditions produced by interference with oxygen transport at the cellular level and probable disruption of electron transport inside the cell. If the level of CO in the blood is high enough that hemoglobin's oxygen-carrying ability is reduced, it can cause tissue hypoxia and permanent damage. The percentage of carboxy-hemoglobin in the blood should be less than 10 %; for this purpose, up to 1000 ppm of production gas will be safe [20]. In this study, the maximum amount of CO produced in FEDBD plasma processing during 6 min with a power of 3.3 W was 3.2 ppm, which is entirely safe.

Electric current: One of the most important influencing factors in electrocution is the intensity of the current. According to the International Electricity Commission standards, the safe current intensity for humans at frequencies between 50 and 60 Hz is equal to 10 mA, and the current intensity that can cause death is 25 mA. This value is determined for direct currents equivalent to 50 mA. According to the ET 213:2007 standard, an electric current of 5 mA for 5 s can cause unwanted muscle contraction. However, there is no risk to the health of living tissue as well as the overall health of the body. In the experiments, the instantaneous peak current produced by the FEDBD plasma device was less than 10 mA. Therefore, physical damage caused by electric current will not affect the health of the patient and the device operator. However, it is recommended to use standard settings in each device to avoid any possible damage [42].

### 3.2 Biometric analysis

**Multi-probe adapter device (MPA-9).** To compare and check skin parameters, skin biometry was performed before plasma processing, immediately after processing, and in the fourth and tenth weeks.

**Fig. 5:**
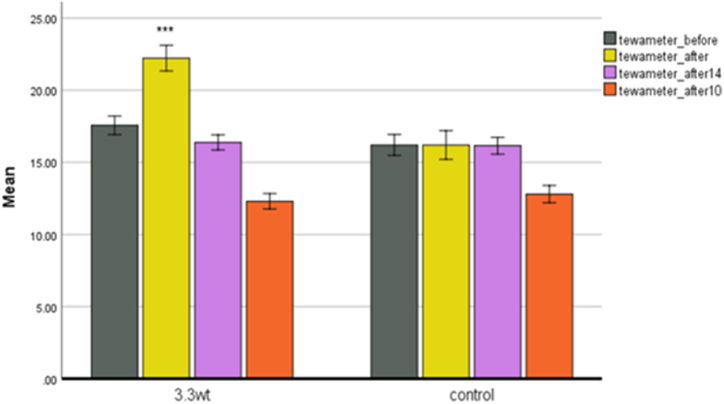
Tewameter changes between two control groups and 3.3 w.

**Tewameter.** Water is constantly evaporated from the skin, part of the body's essential metabolism. Water loss increases with the slightest damage that is not visible to the human eye. Inside the probe, 30 sensors are used to measure this feature so that the results will be very accurate. Also, Tewameter shows evaporation from the surface of the skin. We expect that ageing skin will increase evaporation from its surface and lose more moisture due to the thinning of the epidermal layer and the loss of the protective barrier. Dehydrated skin is more exposed to damage and suffers from spots, dryness, and wrinkles. When we apply plasma to the skin (Fig. 5), we see that immediately after the treatment, due to the heat entering the skin, the evaporation from the surface increases, but over time, the skin is regenerated, and the evaporation from its surface decreases significantly.

For a power of 3.3 W, the gray graph is before plasma processing. The yellow graph shows that after 6 min of plasma processing, the surface's evaporation rate has increased by about 26 % due to the plasma's thermal shock to the skin. The purple chart represents the fourth week of processing. The orange chart shows the 10th week of the experiment, when the skin has had a chance to regenerate, and the amount of evaporation from the surface has decreased, indicating the skin's health. The amount of evaporation from the surface in the control group was higher than in the plasma group in the 10th week.

**Fig. 6:**
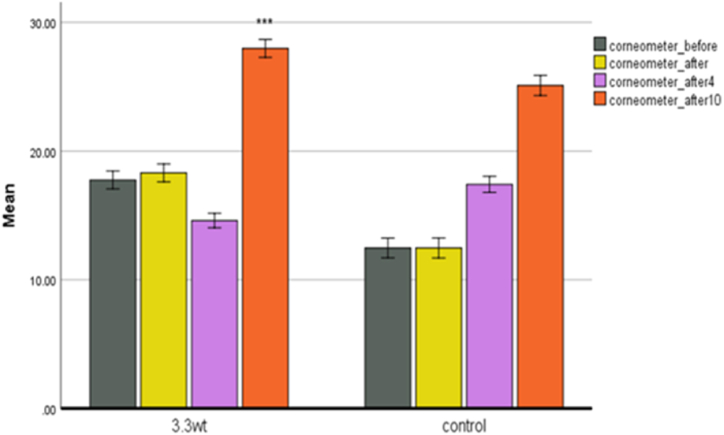
Corneometer changes in two control groups and power of 3.3 w.

**Corneometer.** It indicates skin moisture. Water gives the keratinocyte layer some softness and elasticity. When the skin's water content is normal, the skin looks smooth, soft, elastic, and shiny. Ageing is associated with a physiological process, and the skin loses its ability to retain moisture. In this case, the skin becomes more susceptible to skin infections, including bacteria and fungi, and becomes rough and rough. And he loses his health. Healthy skin is hydrated. Dry skin causes spots, rough texture, and wrinkles. The amount of water on the skin's surface was measured to determine the performance of plasma on its surface. In the plasma group (Fig. 6), a significant increase in skin moisture was observed in the tenth week of treatment. This increase in humidity was also observed in the control group, probably due to environmental conditions.

**Fig. 7:**
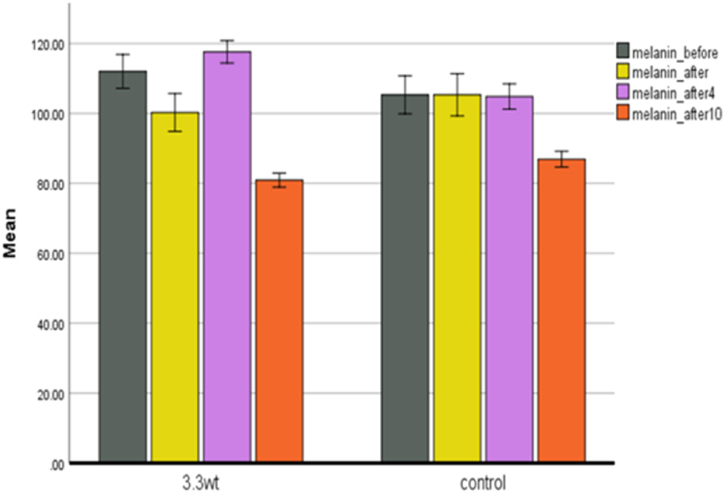
Melanin changes in both control and plasma groups.

**Melanin.** Melanocytes in the basal layer of the epidermis produce melanin pigments. The difference in people's skin color is due to the type of melanin (melanin vs. pheomelanin), the distribution, and the amount of melanin. Also, keratinocytes' size, number, and kind of melanin compartments are effective in different skin colors [27]. In the plasma group (Fig. 7), after one treatment, the amount of melanin decreased by about ten percent, which can be due to the presence of ozone produced in the plasma, which can be effective for lightning the skin in the correct dose. In the fourth week of treatment, we saw a four percent increase in melanin, which probably caused the UV produced by the plasma to affect the melanocytes and cause melanin production. During the recovery period, we saw a 30 % decrease in melanin. Compared to the control group, the skin became lighter, and it is an essential topic for discussing beauty and youth.

**Fig. 8:**
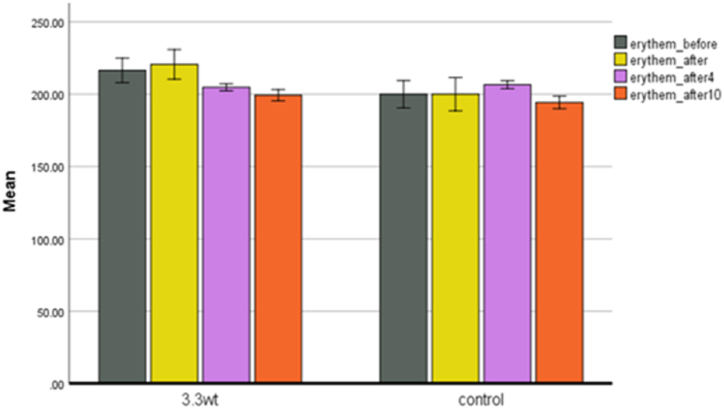
Erythema in both control and plasma groups.

**Erythema.** Erythema is any abnormal skin redness caused by dilation and irritation of superficial capillaries. The increased flow of blood through them gives a reddish color to the skin. If there is a lot of erythema in the skin, its inflammation will increase, and a skin disease will develop afterwards. Where checked using a mexameter probe, and the degree of skin redness or erythema was. Erythema is redness caused by irritation. In the plasma group (Fig. 8), a minimal amount of erythema was observed in the skin immediately after processing, which was caused by thermal shock. The amount of erythema in this group is minimal, and in the later stages of the treatment, it completely disappeared, and even the amount of redness decreased.

**Fig. 9:**
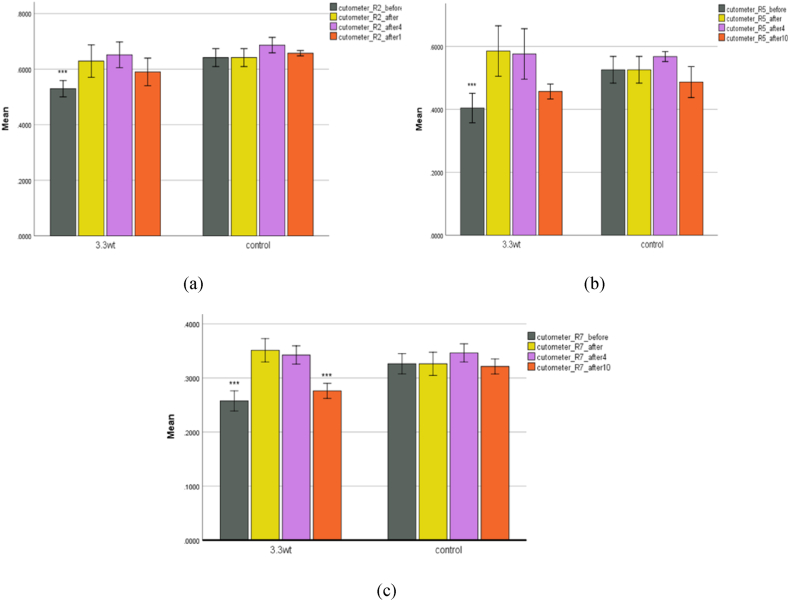
(a) The changes in the R2 index. (b) The number of changes in the index R5. (c) And the amount of R7 index changes in both control and plasma groups.

**Cutometer.** The skin firmness and elasticity degree were measured and recorded using a cutometer. The pre-processing graph in the plasma group (Fig. 9) is significantly lower than in the control group. In the plasma group, the degree of skin stiffness and elasticity in all three indicators increased immediately after treatment, probably due to the formation of edema under the skin. The device measured more stiffness. In the tenth week of the experiment, a slight decrease was observed compared to immediately after processing and the fourth week. During recovery, oxygen and blood supply increase due to various plasma elements and their effect on the skin. Then, the production of keratinocytes and fibroblasts increases, and the skin is regenerated and firmer with collagen synthesis. No significant difference was observed in the control group.

**Skin scanning.** The thickness of the epidermis and dermis is one of the criteria that may be examined in skin rejuvenation. Varied areas of the body have varied skin thicknesses. Furthermore, the skin is shielded from external influences like as cold and heat. The thickening of the epidermal layer reflects its suppleness. The thickness of the epidermis and dermis, as well as their density, were measured in micrometers in this experiment.

**Fig. 10:**
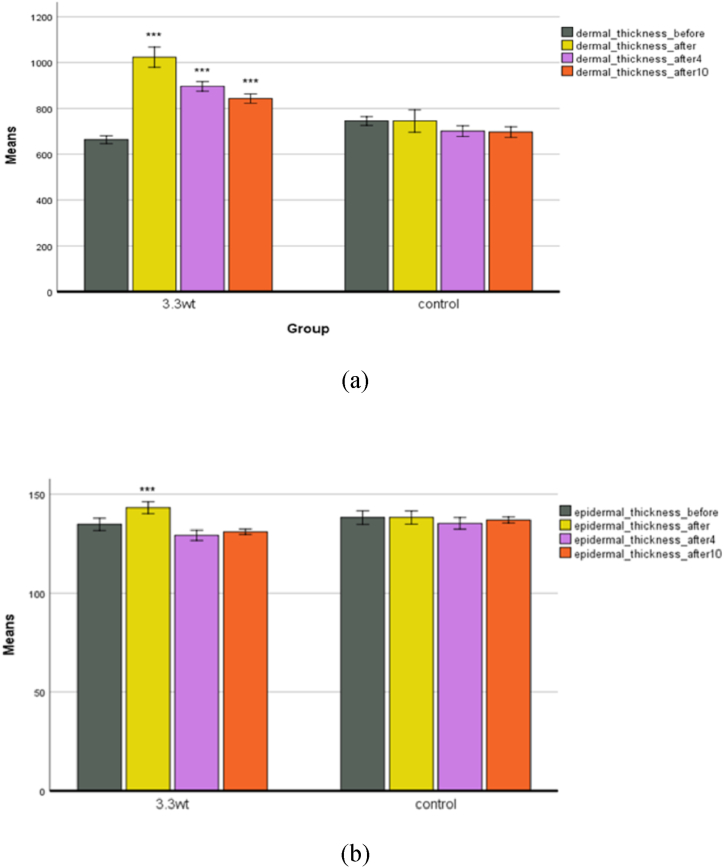
(a) The thickness of the dermis. (b) Epidermal thickness in both control and plasma groups.

The thickness of the epidermis and dermis increased immediately after plasma processing (Fig. 10a and b). This increase in thickness is probably due to the presence of edema. The fluid collects under the tissue, and the other device measures the thickness. But over time, the interstitial fluid is lost, and the thickness of the epidermis and dermis decreases. The thickness of the dermis in the fourth and tenth weeks of the experiment is greater than the thickness of the epidermis. This is because elastin, fibroblasts, and collagen fibers are in the dermis layer [28].

By stimulating the skin, the production and proliferation of these fibers increase, ultimately increasing the skin's thickness, especially in the dermis. Various plasma compounds such as CO, NO, and O3, as well as the created temperature, help in the production and proliferation of keratinocytes and fibroblasts [14,29]. The epidermis and dermis thickness in the control group did not change much and was associated with a slight decrease.

**Fig. 11:**
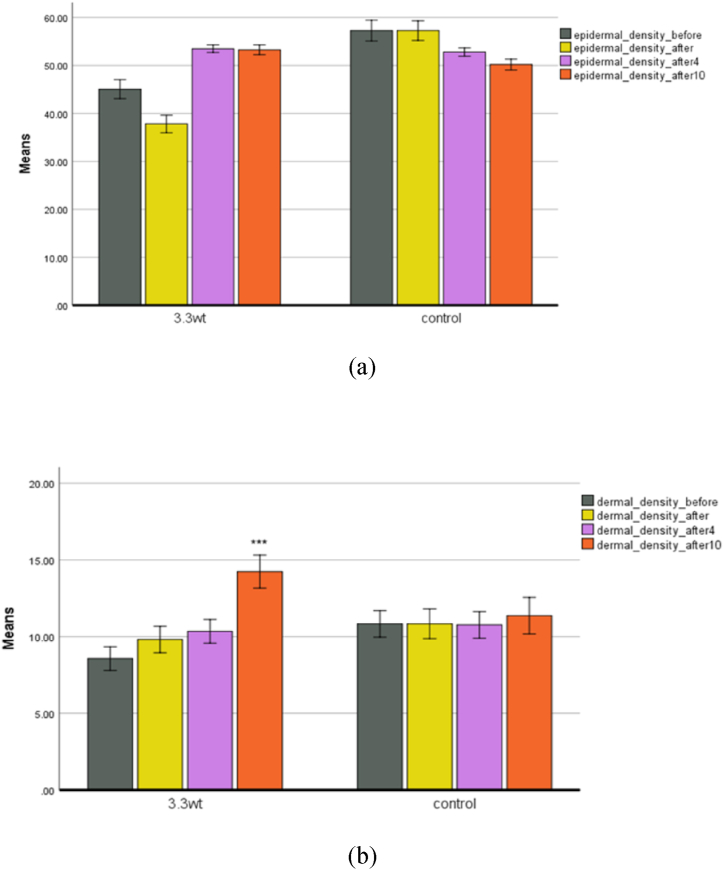
(a) Epidermal density. (b) Dermis density in both control and plasma groups.

**Fig. 12:**
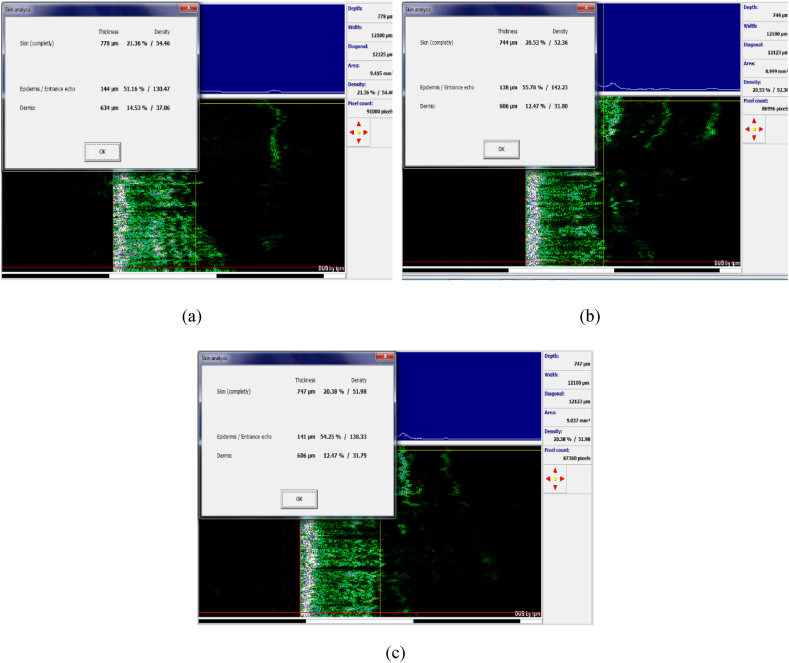
Ultrasound images of the control group. (a) Before the experiment. (b) Forth week. (c) Tenth week.

**Fig. 13:**
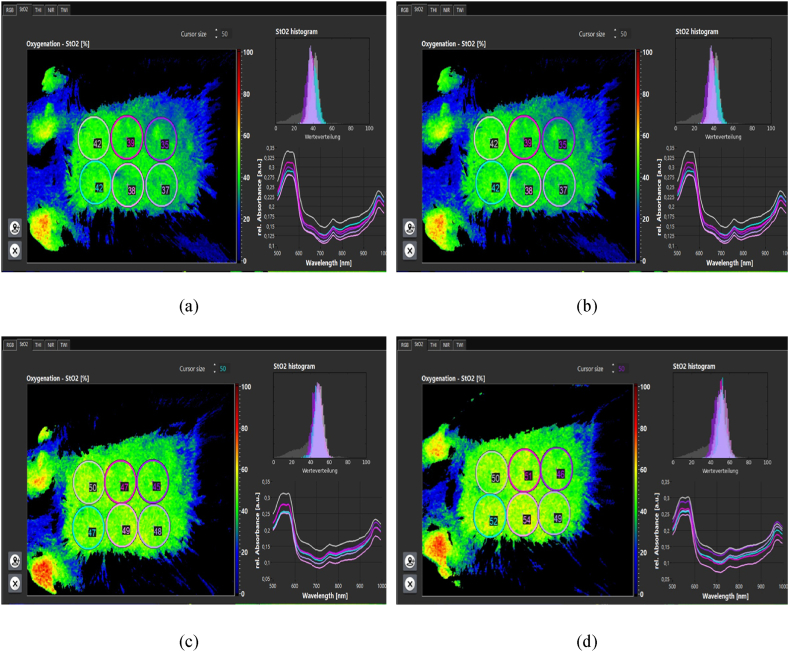
Ultrasound images of the plasma group. (a) Before treatment. (b) Immediately after treatment. (c) Forth week. (d) Tenth week.

The amount of condensation in the epidermis decreased immediately after treatment. When edema occurs (Fig. 11a and b and Fig. 12 a,b,c), the cells become denser and move to the underlying layers, so there is less density in the epidermis layer and more density in the dermis layer. During plasma treatment, with the reduction of edema and the production of different skin cells, including collagen, their density gradually increases in the epidermis and dermis layers. The increase in dermis density (Fig. 13 a,b,c,d) in the plasma group was significant compared to the control group. Epidermal density in the control group decreased over time.

**Evaluation of skin texture.** Cold plasma therapy increases oxygenation through increased perfusion, which promotes angiogenesis. In patients with chronic wounds, it was found that the stimulating effects of plasma increase oxygen and hemoglobin parameters. It also reduces edema, an excess fluid volume in cells or tissues. Reduced edema increases oxygen release to support metabolism and cell growth [30]. Following the increase in oxygen supply and perfusion, the stimulation of fibroblast production increases and increases proportionally to collagen synthesis.

**Fig. 14:**
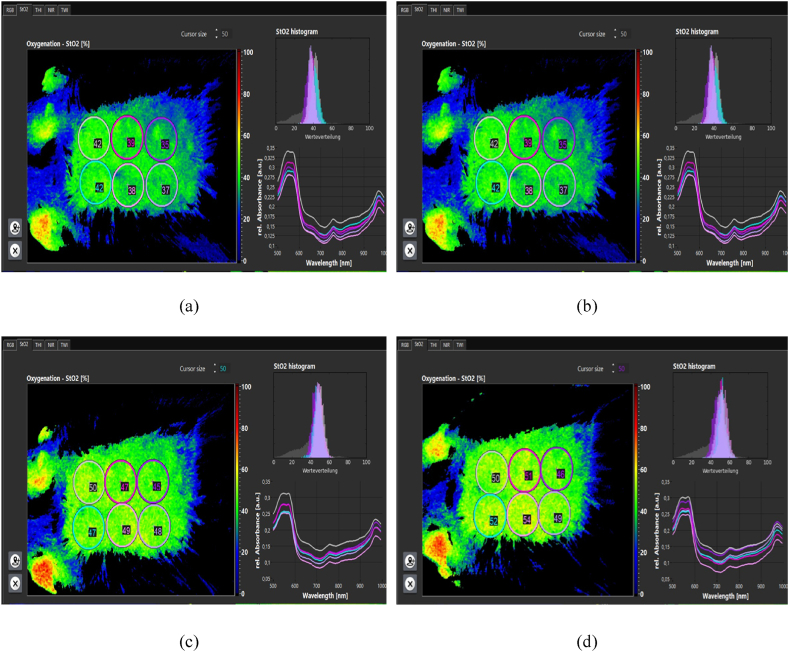
Oxygenation (a) before processing. (b) 30 min after processing. (c) 60 min after processing. (d) 120 min after processing.

**Fig. 15:**
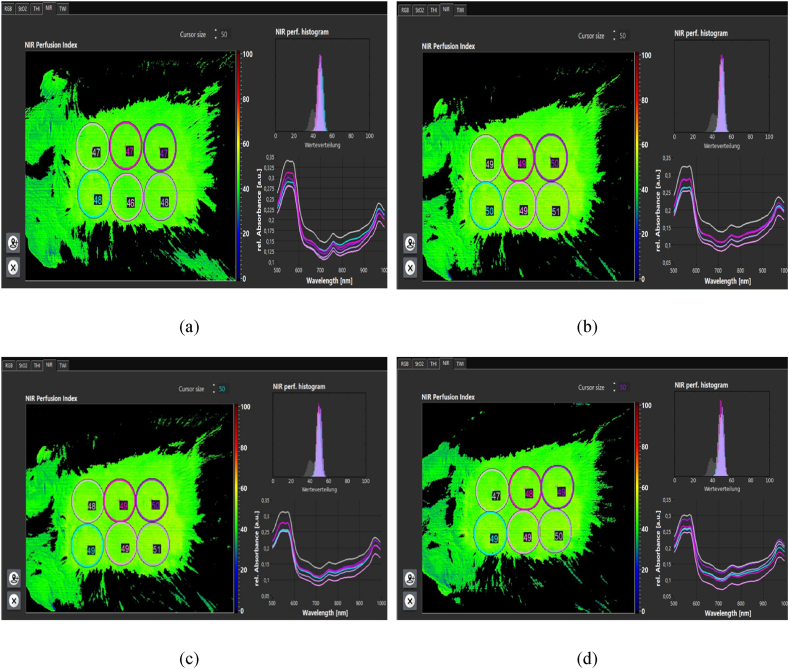
Perfusion. (a) Before processing. (b) 30 min after processing. (c) 60 min after processing. (d) 120 min after processing.

**Fig. 16:**
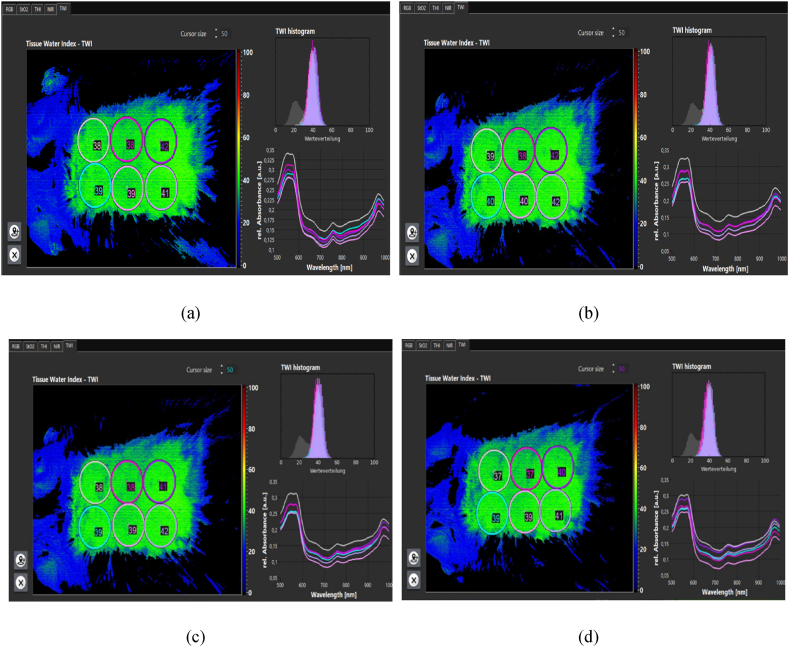
Edema. (a) Before processing. (b) 30 min after processing. (c) 60 min after processing. (d) 120 min after processing.

The amount of tissue oxygenation, perfusion, and edema was measured and recorded using a hyperspectral camera (Fig. 14a,b,c,d). Oxygenation increased after plasma processing. So, 2 h after processing, about a 30 % increase in oxygen supply was observed. The perfusion rate (Fig. 15a,b,c,d) increased by about 6 % up to 2 h after processing. And edema (Fig. 16a,b,c,d) increased by about 2.5 %. Hemoglobin increase was associated with minor changes.

## 4 Discussion

The heat transmission of the gas to the skin causes the plasma to swiftly heat the skin after exiting the device and moving to the treatment region. Heat shocks can boost procollagen type I and procollagen type II production. As a result, they can induce the cells to create more collagen [14]. Unlike ablative technologies (ablative lasers), there is no burning during FEDBD treatment, and which is one of the device's desirable characteristics [31]. Fibroblast activity increases during skin layer rejuvenation [31]. FEDBD works efficiently with little side effects and reduces the risk of unexpected hot spots and scarring. Each plasma parameter interacting with skin tissue has biochemical, biophysical, and biological effects.

The interaction of plasma with air produces a vast number of reactive oxygen and nitrogen species (RONS), including atomic oxygen, ozone (O_3_), nitric oxide (NO), and hydroxyl (OH). These RONs offer a variety of useful qualities, including antimicrobial capabilities and vasodilation [32].

Research shows that NO plays an important role in the skin, especially in the production and proliferation of keratinocytes and fibroblasts [29,33]. Suschek showed that NO produced by plasma could positively affect skin microcirculation due to vasodilation without side effects. Therefore, with the production of various growth factors, cell stimulation, such as the proliferation of fibroblasts, occurs due to the increase in skin blood flow [28,29].

According to additional research, NO normalizes microcirculation, reduces inflammation, and increases bacterial phagocytosis and fibroblast proliferation. As a result of the increased fibroblast cell count, collagen protein synthesis rises, resulting in repair and, hence, rejuvenation [34].

Another important signaling molecule is carbon monoxide (CO). Low concentrations of CO can have beneficial effects on body health. This molecule does not produce toxic side products and is stable. CO is a natural signaler and plays a critical regulatory role as a second messenger in biological systems. It has anti-inflammatory, vasodilating, and antiproliferative properties (Carbone et al., 2018; [21].

It is also employed for a variety of purposes, including organ preservation and tissue ischemia [35]. The carbon monoxide created by plasma interacts with the particles on the skin, cleaning it of lipids and other pollutants. As a result, cleansing the skin's surface can promote blood flow and oxygenation, stimulate fibroblast proliferation, and boost collagen formation. Because plasma and CO have favorable effects in biology on their own, their combination may boost the efficacy of plasma therapies on tissues due to their complementarity [36].

By producing heat, plasma heats the skin. The heat shock produced can enhance procollagen type I and procollagen type II. As a result, raising the temperature encourages cells to generate more collagen [14,22]. The temperature of the tissue should not surpass 40° Celsius. Temperatures between 40 and 50° Celsius might cause localized hyperthermia with cell membranes and molecular structure breakdown. Temperatures above 50 °C cause protein and collagen denaturation and cell fluid evaporation [37].

The reaction to this heat shock stimulates the fibroblast cells in the dermis and stimulates heat shock proteins and collagen synthesis through these cells [22]. Our findings showed that plasma could be effective in increasing the strength and elasticity of the skin due to the proliferation of fibroblast cells and the increase of collagen caused by various plasma compounds, creating a suitable thermal shock with minimal side effects and unpredictable scarring.

Also, increased skin moisture and decreased evaporation from the skin surface were visible. The use of FEDBD plasma had no specific side effects, such as erythema and scarring. Also, an increase in skin hydration and a decrease in evaporation from the skin surface were visible.

Many other research groups worked on plasma skin rejuvenation with complex and expensive devices with complicated equipment that are difficult and expensive to use. Therefore, this article used a device that was easy and inexpensive to operate and did not require anesthesia. This study showed that FEDBD plasma can be used as a modern method to improve skin rejuvenation results.

## 5 Conclusion

As a result of multidisciplinary cooperation in medicine, physics, chemistry, biology, and microbiology, medical plasma has become an innovative and active study topic in recent years. Although many problems remain unresolved, particularly the mechanism of interaction between plasma and live cells/tissues, research to far demonstrate plasma's tremendous medicinal potential. Skin rejuvenation with plasma is one of the innovative treatments that can stimulate skin regeneration while requiring just a brief recovery period and causing no side effects or major tissue damage.

In past research, we used spark plasma to rejuvenate the skin, but this method was associated with pain and minor burns [38]. In this study, we presented a non-invasive and painless method to slow the aging process. The quantitative study of the FEDBD plasma device was used for the first time in rejuvenation. This device does not require anesthesia and is a painless and safe method for beauty and rejuvenation. The most important achievement of this study is maintaining the skin's elasticity, which decreases drastically with age. It also showed that FEDBD plasma can improve skin thickness and density.

Immediately after the treatment, evaporation from the skin surface increased, but evaporation from the surface decreased, and we saw hydration and increased moisture in the skin. Also, the average erythema increased after the treatment due to the plasma heat, but over time, there was no sign of inflammation and redness in the skin. As a result, FEDBD plasma can be a cost-effective, painless, and skin-damaging method for rejuvenation.

## Funding sources

This work was supported by the Skin and Stem Cell Research Center of 10.13039/501100004484Tehran University of Medical Sciences with Grant/Award Number 1400-2-200-54971 and Plasma Medicine Lab of Laser and Plasma Research Institute, Shahid Beheshti University.

## Ethics statement

The authors confirm that all methods were carried out under ARRIVE guidelines and regulations. The ethics committee of the Tehran University of Medical Sciences (IR.TUMS.AEC.1400.050) approved all studies based on the guidelines for the care and use of laboratory animals.

## Data access statement

The datasets used and/or analyzed during the current study are available from the corresponding author upon request.

## CRediT authorship contribution statement

**Parisa Charipoor:** Writing - review & editing, Writing - original draft, Methodology, Investigation, Data curation. **Mohammad Ali Nilforoushzadeh:** Writing - review & editing, Writing - original draft, Visualization, Validation, Supervision, Methodology, Investigation, Funding acquisition, Conceptualization. **Mohammadreza Khani:** Writing - review & editing, Writing - original draft, Visualization, Validation, Supervision, Resources, Project administration, Methodology, Investigation, Funding acquisition, Formal analysis, Data curation, Conceptualization. **Maryam Nouri:** Validation, Methodology, Investigation, Data curation. **Erfan Ghasemi:** Validation, Methodology, Investigation, Formal analysis, Data curation. **Mohammad Amir Amirkhani:** Methodology, Investigation, Data curation. **Mohammad Eftekhari:** Writing - review & editing, Methodology, Investigation, Formal analysis, Conceptualization. **Babak Shokri:** Writing - review & editing, Validation, Methodology, Investigation, Funding acquisition, Formal analysis, Conceptualization.

## Declaration of competing interest

The authors declare that they have no known competing financial interests or personal relationships that could have appeared to influence the work reported in this paper.
